# The Construct and Predictive Validity of the Japanese Version of the Intensive Care Unit Mobility Scale

**DOI:** 10.3390/jcm14165843

**Published:** 2025-08-18

**Authors:** Kohei Tanaka, Nobuto Nakanishi, Shinichi Watanabe, Yasunari Morita, Seiya Sato, Yuko Ono, Kensuke Nakamura, Joji Kotani, Carol L. Hodgson, Hajime Katsukawa

**Affiliations:** 1 Department of Rehabilitation Medicine, Osaka International Medical & Science Center, 2-6-40 Karasugatsuji, Tennoji-ku, Osaka 543-0042, Osaka, Japan; tanaka.kohei30@oim.or.jp; 2 Division of Disaster and Emergency Medicine, Department of Surgery Related, Kobe University Graduate School of Medicine, 7-5-1 Kusunoki-cho, Chuo-ku, Kobe 650-0017, Hyogo, Japan; 3 Department of Physical Therapy, Gifu University of Health Science, 2-92 Higashiuzura, Gifu 500-8281, Gifu, Japan; 4 Department of Emergency and Intensive Care Medicine, National Hospital Organization Nagoya Medical Center, 4-1-1 Sannomaru, Naka-ku, Nagoya 460-0001, Aichi, Japan; 5 Department of Cardiovascular Rehabilitation, National Cerebral and Cardiovascular Center, 6-1 Kishibe-Shimmachi, Suita 564-8565, Osaka, Japan; 6 Australian and New Zealand Intensive Care Research Centre, School of Public Health and Preventive Medicine, Monash University, 553 St. Kilda Rd., Melbourne, VIC 3004, Australia; 7 Intensive Care Unit and Physiotherapy Department, The Alfred Hospital, 55 Commercial Rd., Melbourne, VIC 3004, Australia; 8 Department of Academic Research, Japanese Society for Early Mobilization, 1-2-12 Kudankita, Chiyoda-ku, Tokyo 102-0073, Japan

**Keywords:** intensive care, early mobilization, early rehabilitation, physical assessment, assessment validity

## Abstract

**Background/Objectives**: The increasing emphasis on early mobilization in intensive care units (ICUs) has underscored the need for quick, simple, and reliable tools to assess patients’ mobilization levels. The ICU Mobility Scale (IMS) was developed to address this need and has been translated into a Japanese version. This study aimed to evaluate the construct and predictive validity of the Japanese version of the IMS in critically ill patients. **Methods**: This was a secondary analysis of the EMPICS study, which included patients who stayed in ICUs for at least 48 h. The Japanese version of the IMS and physical function were assessed at ICU discharge. At hospital discharge, outcomes such as walking ability, discharge destination, activities of daily living (ADL) dependency, ICU-acquired weakness, and physical impairment were evaluated. At 90-day follow-up, the presence of post-intensive care syndrome (PICS) was assessed using quality of life scores, and mortality data were collected. Construct and predictive validity were analyzed using Spearman’s rank correlation coefficients, the Mann–Whitney U test, and logistic regression analysis. **Results**: A total of 193 patients (mean age 68.2 years; 65.8% male) were included. The mean IMS score at ICU discharge was 5.6. The IMS score at ICU discharge showed significant correlations with the Barthel Index (ρ = 0.55, *p* = 0.001), Medical Research Council sum score (ρ = 0.45, *p* < 0.001), and grip strength (ρ = 0.44, *p* < 0.001), but not with body weight or sex. Logistic regression analyses demonstrated that a higher IMS score at ICU discharge was significantly associated with better physical outcomes at hospital discharge, a lower incidence of PICS, and reduced 90-day mortality. **Conclusions**: The Japanese version of the IMS demonstrated both construct and predictive validity in ICU patients. It is a useful tool for assessing daily mobilization levels in critical care settings. The findings may not be generalizable to all ICU patients due to the strict eligibility criteria.

## 1. Introduction

Rehabilitation performed in intensive care units (ICUs) has been shown to yield beneficial effects, such as improving physical and cognitive function, reducing ICU length of stay and duration of mechanical ventilation, and lowering the incidence of delirium [1,2,3]. Accordingly, several clinical guidelines recommend proactive early rehabilitation and mobilization [4,5,6]. Various interventions—including mobilization, in-bed cycle ergometry, neuromuscular electrical stimulation, respiratory physical therapy, and dysphagia rehabilitation—are commonly implemented for ICU patients. In clinical practice, early mobilization, such as rolling, sitting, standing, and walking within a few days of ICU admission, is increasingly emphasized. With the growing attention to early mobilization, effective measurement tools are essential for healthcare professionals involved in critical care. The development of a standardized indicator may enable multidisciplinary teams to track mobilization progress and assess the effectiveness of early interventions more efficiently.

A systematic review published in 2012 found that the most commonly used indicators for assessing physical function in studies of early mobilization and rehabilitation in the ICUs were the ability to perform activities such as sitting, standing, and walking [7]. A wide range of indicators had been used in previous studies, making it difficult to compare results across studies. The review therefore highlighted the need for a well-established, standardized indicator. In response, the ICU Mobility Scale (IMS) was developed in 2014 as a tool to quickly, easily, and reliably assess the highest level of mobilization in the ICUs [8]. The IMS is an 11-point ordinal scale ranging from 0 (nothing, i.e., lying in bed and/or passive exercises) to 10 (walking independently without a gait aid). The scale demonstrated strong inter-rater reliability between physical therapists and nurses, as well as construct validity for measuring physical function and predictive validity for mortality and discharge outcomes [8,9]. The IMS is now widely used in clinical practice and is considered a standard tool for assessing mobilization levels in critical care settings.

The Japanese version of the IMS was developed by medical professionals in Japan, in collaboration with Hodgson, the developer of the original version [10]. The Japanese version was created through a rigorous, iterative translation process. First, the original IMS was translated into Japanese. Multiple professionals then reviewed the translation for consistency with the English version and made necessary revisions. A back-translated version was subsequently created and reviewed by the original author. This process continued until the original author no longer identified any issues. The Japanese version of the IMS has demonstrated feasibility and strong inter-rater reliability [11]. However, its validity has not yet been evaluated. To ensure that translated assessment tools accurately reflect the concepts of the original version, it is important to assess their validity across different languages and cultural contexts [12]. We hypothesize that the Japanese version, like the original IMS, correlates with physical function and can predict clinical outcomes. The aim of this study was to evaluate the construct and predictive validity of the Japanese version of the IMS in critically ill patients.

## 2. Materials and Methods

### 2.1. Study Design, Settings, and Patients

This is a secondary analysis of the EMPICS study (association between the early mobilization and psychiatric symptoms, UMIN ID: UMIN000036503, URL: https://center6.umin.ac.jp/cgi-open-bin/ctr_e/ctr_view.cgi?recptno=R000041241 (accessed on 17 July 2025)) [13]. The EMPICS study was approved by the Ethics Committee of Nagoya Medical Center (Approval No. 2018093; approved on 15 May 2019) and by the ethics committees of eight other hospitals: Hitachi General Hospital, Nagasaki University Hospital, Fukuyama City Hospital, Naha City Hospital, Yuuai Medical Center, Tokushukai General Hospital, Showa University Hospital, and Tokyo Women’s Medical University Hospital. Patients were eligible for inclusion if they had stayed in the ICUs for at least 48 h between June and December 2019. One of the objectives of this secondary analysis was to investigate the association between the Japanese version of the IMS and physical dysfunction at hospital discharge, defined as a Barthel Index (BI) score of less than 70. To ensure the accuracy of this assessment, patients with poor physical function at ICU admission needed to be excluded. The exclusion criteria were as follows: age under 18 years, inability to walk even with a cane, BI score < 70 at ICU admission, presence of neurological disease, communication difficulties, withdrawal of treatment, or death during ICU stay.

### 2.2. IMS Assessment

The IMS is an 11-point ordinal scale that evaluates the highest level of patient mobilization, with the following scoring criteria. Score 0: lying/passive exercises in bed, score 1: any activity in bed, score 2: passive transfer to chair, score 3: sitting over edge of bed, score 4: standing, score 5: transfer from bed to chair, score 6: marching on the spot, score 7: walking with assistance from 2 or more people, score 8: walking with assistance from 1 person, score 9: independent walking with a walking aid, and score 10: independent ambulation. Physical therapists assessed patients at ICU discharge using the Japanese version of the IMS. The Japanese version of the IMS is publicly available on the website of the Japanese Society for Early Mobilization (URL: https://www.rishou.org/activity-new/scaletool#/ (accessed on 17 July 2025)).

### 2.3. Other Data Collection

Physical assessments, including the BI, Medical Research Council (MRC) sum score, and grip strength, were conducted at ICU discharge and hospital discharge. In addition to these assessments, walking ability (with or without assistance) and the Short Physical Performance Battery were evaluated at hospital discharge. Discharge destination, categorized as home or other locations such as a rehabilitation ward or nursing care facility, was also recorded. At 90 days after discharge, the EuroQol 5-Dimensions 5-Levels (EQ-5D-5L) visual analog scale and mortality data were collected. The EQ-5D-5L was converted to a ratio scale anchored at 0 (death) and 1 (full health) using Japanese population-specific coefficients derived from the five dimensions [14].

### 2.4. Construct Validity

Construct validity was assessed by examining the relationships between the Japanese version of the IMS score at ICU discharge and physical function indicators at ICU discharge (BI, MRC sum score, and grip strength), which are theoretically expected to be related. In contrast, relationships with unrelated variables, such as body weight and sex, were also examined to confirm discriminant validity.

### 2.5. Predictive Validity

Predictive validity was evaluated based on the hypothesis that the Japanese version of the IMS score at ICU discharge would be associated with physical function at hospital discharge, quality of life (QOL) at 90-day follow-up, and 90-day mortality. Physical function at hospital discharge was assessed using the following indicators: walking ability (with or without assistance), discharge destination (home or another facility), activities of daily living (ADL) dependency (defined as a Barthel Index score < 70 [15]), ICU-acquired weakness (ICU-AW) (defined as an MRC sum score < 48 [16]), and physical impairment (defined as a Short Physical Performance Battery score < 12 [17]). QOL was assessed using the EQ-5D-5L, and post-intensive care syndrome (PICS) was defined as an EQ-5D-5L score < 0.72 [18].

### 2.6. Floor and Ceiling Effects

Floor and ceiling effects were assessed by calculating the percentage of participants who scored the minimum (0) and maximum (10) values on the Japanese version of the IMS at ICU discharge. A proportion below 15% for either extreme was considered indicative of an acceptable level of distributional bias [19].

### 2.7. Statistical Analysis

Continuous variables with a normal distribution are presented as mean ± standard deviation (SD), while categorical variables are expressed as number and percentage (%). The sample size was predetermined, as this study involved a secondary analysis of existing data collected in the original study [13]. As a result, an a priori sample size calculation was not feasible for this study. The correlations between the Japanese version of the IMS score at ICU discharge and physical function indicators, as well as body weight, were assessed using Spearman’s rank correlation coefficients. Additionally, we created locally estimated scatterplot smoothing (LOESS) curves—a type of nonlinear regression analysis—to visually confirm the distribution. Differences in the IMS scores between male and female patients were evaluated using the Mann–Whitney U test. Predictive validity was examined using logistic regression analyses, with the Japanese version of the IMS score at ICU discharge as the explanatory variable. The following outcomes were used as dependent variables in separate regression models: walking ability, discharge destination, ADL dependency, ICU-AW, physical impairment, presence of PICS, and 90-day mortality. Potential confounders including age, sex, body mass index, Acute Physiology and Chronic Health Evaluation II (APACHE II) score, Charlson Comorbidity Index, and duration of mechanical ventilation were included as covariates in the logistic regression analyses. These covariates were selected a priori based on previous literature and biological plausibility. Results are presented as odds ratios (ORs) with 95% confidence intervals (CIs) and corresponding *p*-values. The Hosmer–Lemeshow test was used to evaluate the goodness of fit for each logistic regression model. Cut-off values for the Japanese version of the IMS at ICU discharge were determined using receiver operating characteristic (ROC) curve analysis and the Youden index. Sensitivity, specificity, and the area under the curve (AUC) for each analysis are also reported. Statistical significance was defined as a *p*-value ≤ 0.05. All analyses were performed using R version 4.2.2 (The R Foundation for Statistical Computing, Vienna, Austria).

## 3. Results

**Figure 1:**
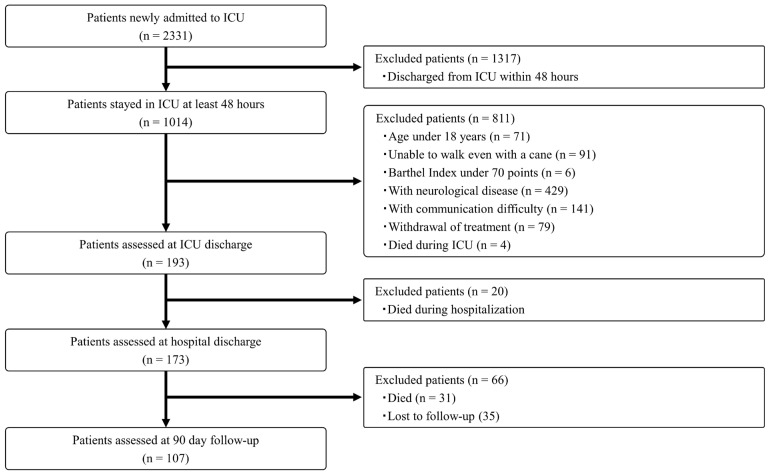
Patient flow diagram.

During the study period, a total of 2331 patients were newly admitted to the ICUs. Of these, 2128 patients were excluded based on the predefined analytical protocol. Assessments were completed for 193 patients at ICU discharge, 173 patients at hospital discharge, and 107 patients at the 90-day follow-up. The patient flow diagram is presented in Figure 1, and the clinical characteristics of the enrolled patients are summarized in Table 1. The mean age of participants was 68.2 years, and 127 patients (65.8%) were male. The mean APACHE II score was 19.9. The average length of ICU stay was 7.7 days, and the mean hospital stay was 37.7 days.

**Figure 2:**
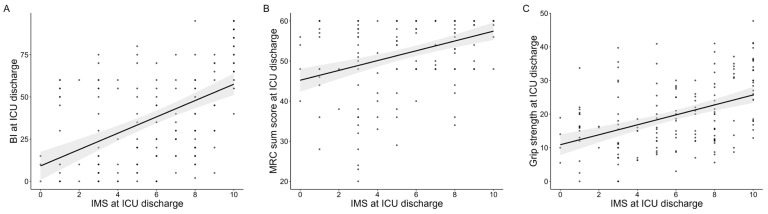
Correlations of the Japanese version of the IMS value with physical functions at ICU discharge. Correlations of the IMS value at ICU discharge with BI (**A**), MRC-sum score (**B**), and grip strength (**C**). The gray area indicates the 95% confidence interval. IMS: ICU mobility scale, ICUs: intensive care units, MRC: medical research council.

**Figure A1:**
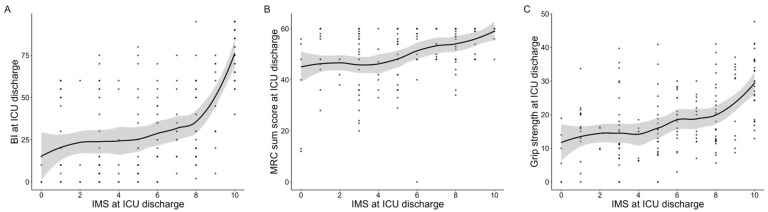
Scatter plot and LOESS curves of the Japanese version of the IMS value with physical functions at ICU discharge. Scatter plot and LOESS curves for the IMS value at ICU discharge with BI (**A**), MRC-sum score (**B**), and grip strength (**C**). The gray area indicates the 95% confidence interval. LOESS: locally estimated scatterplot smoothing, IMS: ICU mobility scale, ICUs: intensive care units, MRC: medical research council.

The results of the correlation analyses are presented in Figure 2. The IMS value at ICU discharge showed statistically significant correlations with the BI (ρ = 0.55, *p* = 0.001), the MRC sum score (ρ = 0.45, *p* < 0.001), and grip strength (ρ = 0.44, *p* < 0.001). In contrast, the IMS value was not significantly correlated with body weight (ρ = 0.08, *p* = 0.920), and there was no significant difference between male and female patients (male: 5.4 vs. female: 5.2, *p* = 0.560). The LOESS curves did not show clear nonlinear distributions (Figure A1).

Logistic regression analyses showed that the higher score of the IMS at ICU discharge was significantly associated with following clinical variables at hospital discharge: walking without assistance (OR: 1.19, 95% CI: 1.00–1.43, *p* = 0.050), discharge to home (OR: 1.16, 95% CI: 1.01–1.34, *p* = 0.033), ADL dependence (OR: 0.85, 95% CI: 0.70–1.00, *p* = 0.050), ICU-AW (OR: 0.77 95% CI: 0.62–0.95, *p* = 0.018), and physical impairment (OR: 0.85, 95% CI: 0.72–0.99, *p* = 0.036). It was also significantly associated with PICS symptoms at 90 days (OR: 0.80, 95% CI: 0.67–0.94, *p* = 0.007), and survival rate at 90 days (OR: 0.83, 95% CI: 0.72–0.96, *p* = 0.014) (Table 2). The results of ROC analyses are shown in Table 3. ROC curves for each analysis are presented in Figure A1. The cut-off values of the IMS were 5.5 for walking without assistance (sensitivity, 0.64; specificity, 0.75; AUC, 0.69), discharge to home (sensitivity, 0.63; specificity, 0.64; AUC, 0.66), ADL dependence (sensitivity, 0.63; specificity, 0.63; AUC, 0.67), ICU-AW (sensitivity, 0.94; specificity, 0.63; AUC, 0.76), and 90-day mortality (sensitivity, 0.65; specificity, 0.58; AUC, 0.64). The cut-off values of the IMS were 6.5 for physical impairment (sensitivity, 0.66; specificity, 0.65; AUC, 0.68) and PICS symptoms (sensitivity, 0.75; specificity, 0.63; AUC, 0.71).

**Figure A2:**
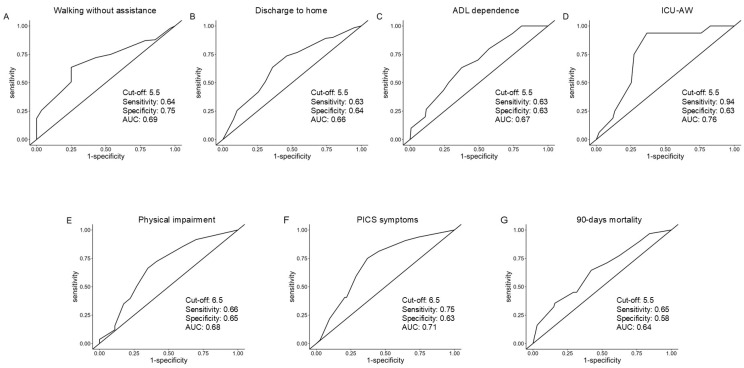
Receiver operator characteristic curves demonstrating the predictive utilities of the Japanese version of the IMS value at ICU discharge. Each graph shows the results of the ROC analysis for walking without assistance (**A**), discharge to home (**B**), ADL dependence (**C**), ICU-AW (**D**), physical impairment (**E**), PICS symptoms (**F**), 90-day mortality (**G**). IMS: ICU mobility scale, ICUs: intensive care units, ROC: receiver operating characteristic, ADL: activities of daily living, ICU-AW: ICU-acquired weakness, PICS: post-intensive care syndrome.

The frequency distribution of the IMS scores is presented in Figure A2. Six patients (3.0%) scored the minimum value of 0, while 28 patients (14.1%) achieved the maximum score of 10. The mean IMS score at ICU discharge was 5.3 ± 3.2. The mean ± standard deviation values fell within the full range of IMS scores (0 to 10).

## 4. Discussion

Using multicenter observational data, this study evaluated the validity of the Japanese version of the IMS as a tool for assessing physical function. The IMS was significantly correlated with muscle strength and physical function at ICU discharge. Furthermore, IMS at ICU discharge predicted the occurrence of ICU-AW at hospital discharge, as well as the incidence of PICS and mortality at 90 days. These findings demonstrate that the Japanese version of the IMS possesses both construct and predictive validity for assessing the mobilization level of ICU patients.

The IMS was developed to provide a structured method for assessing patient mobility levels in ICUs, aiming to facilitate the monitoring of recovery, quantify mobilization levels, and enable comparisons across studies [8]. It allows for rapid assessment of physical function without the need for specialized instruments. The Japanese version of the IMS was created through a rigorous academic process involving repeated translation and revision. A previous study validated the original IMS by statistically analyzing its association with physical function at ICU discharge, as well as its predictive ability for ICU-AW at hospital discharge and 90-day mortality [9]. Building on the analytical approaches of that study, we examined the construct and predictive validity of the Japanese version of the IMS. The Japanese version of the IMS showed statistically significant associations with physical function parameters and clinical outcomes, both of which had been hypothesized a priori. These findings support the validity of the Japanese version and suggest that the original instrument was accurately translated and can be appropriately applied in Japanese ICU settings.

The Japanese version of the IMS has already been utilized for both clinical and research purposes in ICUs in Japan. It has served as an outcome measure to evaluate the effects of early rehabilitation in randomized controlled trials and as an indicator for comparing mobilization levels across study groups or between different facilities [20,21,22]. However, prior to this study, no research had been conducted to directly support the validity of the Japanese version. Consequently, previous studies employing the Japanese version of the IMS relied on the validity of the original version as surrogate evidence. The present findings provide empirical support for the validity of the Japanese version of the IMS, which can now serve as a foundation for future research. Accordingly, the Japanese version of the IMS is expected to be increasingly utilized as a valid and important tool for assessing mobilization levels in early rehabilitation among the Japanese ICU population.

The limitations of this study are outlined below. Firstly, this study included patients from a variety of backgrounds. There would be differences in the general progress of rehabilitation among patients who underwent scheduled surgery, emergency surgery, or were admitted to the ICUs following a sudden change. The Japanese version of the IMS values and outcomes were probably confounded by varying patient backgrounds. However, the results of this study suggest that the Japanese version of the IMS assessment is suitable for patients who are potentially being admitted to the ICUs. Second, a priori sample size calculation was not performed, as this was a secondary analysis. While approximately 10 events per variable are generally recommended for logistic regression analyses, this criterion could not be fully ensured in the present study [23]. This study had a relatively small sample size compared to the number typically recommended for performing regression analyses. However, the AUC in the ROC analysis was approximately 0.7, suggesting that the model demonstrated fair predictive performance. Another limitation is the concern about selection bias. We included patients who stayed in the ICUs for more than 48 h and excluded patients based on strict exclusion criteria (e.g., presence of neurological disease and death during ICU stay). Patients with poor physical function prior to ICU admission—such as those who were non-independent in ADL or ambulation—were also excluded. Given that the final sample size comprised only 8.3% of initial screening, selection bias is a concern. The results of this study should be interpreted with caution in terms of generalizability. Further research is warranted to evaluate the validity of the Japanese version of the IMS in populations with limited physical function prior to ICU admission.

## 5. Conclusions

The Japanese version of the IMS demonstrated both construct and predictive validity in ICU patients. Consistent with the original version, the Japanese version of the IMS is a useful tool for assessing daily mobilization levels in the ICU setting.

## Figures and Tables

**Table 1:** Clinical characteristics of enrolled patients.

Variables	Total (n = 193)
Age	68.2 (14.7)
Sex	
Male (%)	127 (65.8)
Female (%)	66 (34.2)
Body mass index	23.1 (4.3)
ICU admission diagnosis	
Cardiovascular disease	73 (37.8%)
Sepsis	31 (16.1%)
Acute respiratory failure	28 (14.5%)
Gastrointestinal surgery	25 (13.0%)
Others	36 (18.7%)
APACHE II score	19.9 (8.2)
SOFA score	7.1 (4.02)
Mechanical ventilation (%)	122 (63.2)
Artificial dialysis (%)	42 (21.8)
Charlson comorbidity index	2.1 (2.1)
Length of mechanically ventilated (day)	5.4 (15.1)
Length of ICU stay (days)	7.3 (8.2)
Length of hospital stay (day)	37.7 (33.2)
Hospital mortality	
Time to rehabilitation (day)	2.2 (5.8)
Japanese version of the IMS at ICU discharge	5.6 (3.1)
Japanese version of the IMS maximum in ICUs	6.0 (2.9)
Physical function at ICU discharge	
BI	35.5 (28.3)
MRC sum score	51.5 (10.8)
Grip strength	19.0 (10.2)
Physical function at hospital discharge	
Barthel index	82.6 (26.3)
MRC sum score	55.2 (8.0)
Grip strength	22.7 (10.4)
Short physical performance battery	9.0 (4.01)
Walking without assistance (%)	144 (83.2)
Home discharge (%)	133 (76.9)
90-day follow-up	
EuroQol-5 Dimensions-5 Levels	0.77 (0.27)
90-day mortality	31 (18.5)

The continuous variables were presented as the mean ± standard deviation, and the categorical variables were presented as number (%). ICUs: intensive care units, APACHE II: acute physiology and chronic health disease classification system II, SOFA: sequential organ failure assessment, IMS: ICU mobility scale, MRC: medical research council.

**Table 2:** Logistic regression analysis for clinical outcome.

	N	Odds Ratio	95% CI		*p* Value	Hosmer–Lemeshow
			Lower	Upper		
At hospital discharge						
Walking without assistance	168	1.19	1.00	1.43	0.050	0.88
Discharge to home	168	1.16	1.01	1.34	0.033	0.66
ADL dependence	166	0.85	0.70	1.00	0.050	0.24
ICU-AW	166	0.77	0.62	0.95	0.018	0.17
Physical impairment	146	0.85	0.72	0.99	0.036	0.51
At 90-day follow-up						
PICS	105	0.80	0.67	0.94	0.007	0.75
Mortality	158	0.83	0.72	0.96	0.014	0.03

Logistic regression analyses were performed, using the Japanese version of the IMS as the independent variable and each clinical outcome as the dependent variable. The results of the Japanese version of the IMS for each regression analysis are summarized in Table 2. All regression models were adjusted for age, sex, body mass index, APACHE II score, Charlson Comorbidity Index, and mechanical ventilation duration. ADL: Activities of daily living, ICU-AW: ICU-acquired weakness, PICS: post-intensive care syndrome, 95% CI: 95% confidence interval.

**Table 3:** Cut-off value of the Japanese version of the IMS at ICU discharge predicting clinical outcomes.

	Cut-off Value	Sensitivity	Specificity	Area Under the Curve
At hospital discharge				
Walking without assistance	5.5	0.64	0.75	0.69
Discharge to home	5.5	0.63	0.64	0.66
ADL dependence	5.5	0.63	0.63	0.67
ICU-AW	5.5	0.94	0.63	0.76
Physical impairment	6.5	0.66	0.65	0.68
At 90-day follow-up				
PICS	6.5	0.75	0.63	0.71
Mortality	5.5	0.65	0.58	0.64

Cut-off values of the Japanese version of the IMS for each clinical outcome were determined using ROC curve analysis with the Youden index. IMS: ICU mobility scale, ICUs: intensive care units, ADL: Activities of daily living, ICU-AW: ICU-acquired weakness, PICS: post-intensive care syndrome.

**Figure A3:**
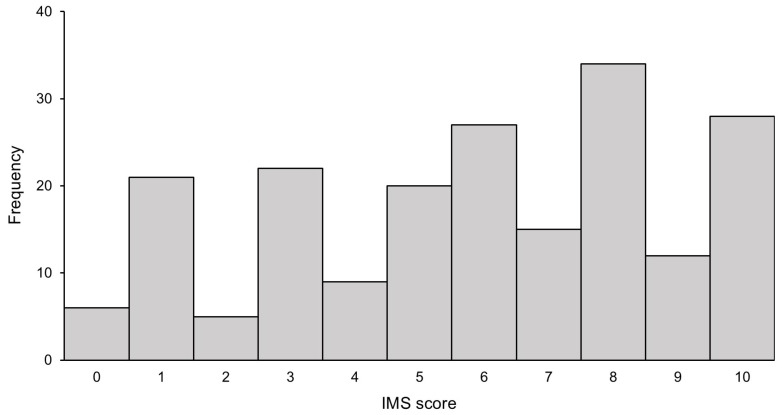
The frequency distribution of enrolled patients based the Japanese version of the IMS value at ICU discharge. The bar chart shows the number of patients according to their Japanese version of the IMS score. IMS: ICU mobility scale, ICUs: intensive care units.

## Data Availability

The data presented in this study are not publicly available, but they can be accessed upon reasonable request to the corresponding author.

## Abbreviations

The following abbreviations are used in this manuscript:

ICUs	Intensive care units
IMS	ICU mobility scale
BI	Barthel index
MRC	Medical research council
EQ-5D-5L	EuroQol 5-Dimensions 5-Levels
QOL	Quality of life
ADL	Activities of daily living
ICU-AW	ICU-acquired weakness
PICS	Post-intensive care syndrome
SD	Standard deviation
LOESS	Locally estimated scatterplot smoothing
APACHE II	Acute physiology and chronic health evaluation II
OR	Odds ratios
CI	Confidence intervals
ROC	Receiver operating characteristic
AUC	Area under the curve
